# Temporal transcriptional patterns of cyanophage genes suggest synchronized infection of cyanobacteria in the oceans

**DOI:** 10.1186/s40168-020-00842-9

**Published:** 2020-05-19

**Authors:** Yue Chen, Qinglu Zeng

**Affiliations:** 1 Department of Ocean Science, The Hong Kong University of Science and Technology, Clear Water Bay, Hong Kong, China; 2 Division of Life Science, The Hong Kong University of Science and Technology, Clear Water Bay, Hong Kong, China; 3 HKUST Shenzhen Research Institute, Shenzhen, China; 4 Hong Kong Branch of Southern Marine Science and Engineering Guangdong Laboratory (Guangzhou), The Hong Kong University of Science and Technology, Clear Water Bay, Hong Kong, China

**Keywords:** Cyanobacterium, Cyanophage, Diurnal rhythm, Light-dark cycle, Metatranscriptomics

## Abstract

**Background:**

Based on the peak expression times during infection, early, middle, and late genes have been characterized in viruses (cyanophages) that infect the unicellular cyanobacterium *Prochlorococcus*. Laboratory experiments show that some cyanophages can only replicate in the light and thus exhibit diurnal infection rhythms under light-dark cycles. Field evidence also suggests synchronized infection of *Prochlorococcus* by cyanophages in the oceans, which should result in progressive expression of cyanophage early, middle, and late genes. However, distinct temporal expression patterns have not been observed in cyanophage field populations.

**Results:**

In this study, we reanalyzed a previous metatranscriptomic dataset collected in the North Pacific Subtropical Gyre. In this dataset, it was previously shown that aggregate transcripts from cyanophage scaffolds display diurnal transcriptional rhythms with transcript abundances decreasing at night. By mapping metatranscriptomic reads to individual viral genes, we identified periodically expressed genes from putative viruses infecting the cyanobacteria *Prochlorococcus* and *Synechococcus*, heterotrophic bacteria, and algae. Of the 41 cyanophage genes, 35 were from cyanomyoviruses. We grouped the periodically expressed cyanomyovirus genes into early, middle, and late genes based on the conserved temporal expression patterns of their orthologs in cyanomyovirus laboratory cultures. We found that the peak expression times of late genes in cyanophage field populations were significantly later than those of early and middle genes, which were similar to the temporal expression patterns of synchronized cyanophage laboratory cultures.

**Conclusions:**

The significantly later peak expression times of late genes in cyanomyovirus field populations suggest that cyanophage infection of *Prochlorococcus* is synchronized in the North Pacific Subtropical Gyre. The night-time peak expression of late genes also suggests synchronized lysis of *Prochlorococcus* at night, which might result in synchronized release of dissolved organic matter to the marine food web.

Video abstract.

## Background

To adapt to the earth’s daily light-dark (diel) cycle, diurnal rhythms of gene expression, metabolism, and behavior have been shown in all three domains of life [11], including cyanobacteria [7]. The unicellular picocyanobacteria *Prochlorococcus* and *Synechococcus* are the major primary producers in the oceans [13, 33, 38]. In the North Pacific Subtropical Gyre, rhythmic transcriptional patterns have been observed in *Prochlorococcus* and several co-occurring heterotrophic bacterial groups [32]. The diurnal transcriptional rhythm of marine microbial populations is suggested to be a result of metabolic coupling between *Prochlorococcus* and heterotrophic bacteria, which may influence matter and energy transformation in the sea [32].

*Prochlorococcus* and *Synechococcus* are frequently infected by lytic double-stranded DNA viruses (cyanophages) [27, 43], which rely on the photosynthetic energy of host cells to replicate [23, 25, 40]. Cyanophage strains exhibiting three classes of diel-dependent life history traits (“diel traits”) have been characterized [24]: (1) no adsorption to the host cells in the dark [9, 15, 16, 24], (2) adsorption in the dark but no replication, and (3) adsorption and replication in the dark, but with a smaller burst size than in the light. Regardless of the trait, cyanophage transcripts are more abundant during infection in the light than those in the dark, resulting in a diurnal transcriptional rhythm under light-dark cycles [24]. The diurnal rhythm of cyanophage transcripts is likely caused by the photosynthetic activity of cyanobacterial host cells, since an inhibitor of photosynthetic electron flow can also reduce cyanophage transcripts in the light [24].

The three classes of diel traits indicate that cyanophages that are unable to adsorb or replicate in the dark can only initiate the infection process in the light [24]. Therefore, in marine environments, we hypothesized that the infection process of these cyanophages should be synchronized to the light-dark cycle. Indeed, there is field evidence to suggest synchronized infection of *Prochlorococcus* and *Synechococcus* in the oceans [6, 37, 51]. In the subtropical Pacific Ocean, the mortality rate of *Prochlorococcus* was found to be synchronized to the light-dark cycle [37]. The highest mortality rate appeared at night and could be caused by synchronized cyanophage burst at night; however, the synchronized mortality rate could also be explained by synchronized grazing of protists [37]. In the Indian Ocean, infectious cyanophages from surface waters were isolated using *Synechococcus* sp. WH7803 as the host and were shown to have the highest abundance at night [6]. Yoshida et al. reasoned that synchronized infection should result in an increase of viral transcripts followed by an increase of virus particles [51]. To test this, Yoshida et al. [51] conducted metagenomic and metatranscriptomic studies using surface seawater from Osaka Bay off Japan, and observed that viruses with relatively high transcript abundances generally increased their genome abundance ranks at a later time point. However, a higher genome abundance rank does not necessarily mean a higher virus abundance. More critically, both the Indian Ocean study [6] and the Osaka Bay study [51] were conducted only for 1 day, so it is uncertain whether the patterns shown in both studies are diurnal rhythms that can repeat for several days.

Similar to bacteriophages [30], cyanophage infection relies on a robust temporal transcriptional program [5, 10, 21, 22, 46]. According to the peak expression times during infection, cyanophage genes can be categorized into early, middle, and late genes that play different roles [10, 21, 22, 46]. For example, early genes might be involved in modifying the host RNA polymerase, middle genes are related to phage genome replication, and late genes mainly encode structural proteins for phage capsid assembly [10, 21]. In unsynchronized infections, early, middle, and late genes are being transcribed at any time, and should all reach their peak expression levels at around sunset due to the lack of photosynthetic activity of the host cells at night [24]. On the other hand, in synchronized infections, cyanophages initiate the infection process shortly after sunrise and the peak expression times of early, middle, and late genes should be discrete.

A metatranscriptomic study conducted by Aylward et al. in the North Pacific Subtropical Gyre revealed diurnal transcriptional rhythms of cyanophages, with peak transcript abundances at around sunset [1]. In this study, the authors sequenced viral metagenomes and metatranscriptomes (in the microbial cellular size fractions) within a coherent water mass at a depth of 15 m in the North Pacific Subtropical Gyre every 4 h over 8 days (July 27–31 and August 1–3, 2015) [1]. By mapping viral transcripts to the 170 viral scaffolds assembled from viral metagenomes, they identified 26 viral scaffolds with rhythmic aggregate transcript abundances, including 17 scaffolds similar to cyanophage genomes, one similar to *Pelagibacter* phage (pelagiphage), and several unclassified ones [1]. However, it was not shown in this study whether individual viral genes in a scaffold show diurnal transcriptional rhythms, as the combined transcripts mapped to all the genes in a scaffold were used for diel periodicity analyses [1].

Here, we explored the synchronization of cyanophage infection in marine environments on a gene-by-gene (rather than total abundance) basis. To do so, we re-examined the expression of cyanophage genes from the metatranscriptomic study conducted by Aylward et al. [1]. Building upon Aylward’s finding of peaks in total transcriptional activity, we found that the early, middle, and late genes of natural cyanophage populations show distinct peak expression times, suggesting that cyanophage infection is synchronized in the surface ocean. Since *Prochlorococcus* is the dominant primary producer in the North Pacific Subtropical Gyre [32], synchronized infection of *Prochlorococcus* may affect carbon cycling in this region.

## Results

### Viral genes in the North Pacific Subtropical Gyre show diurnal transcriptional rhythms

**Fig. 1:**
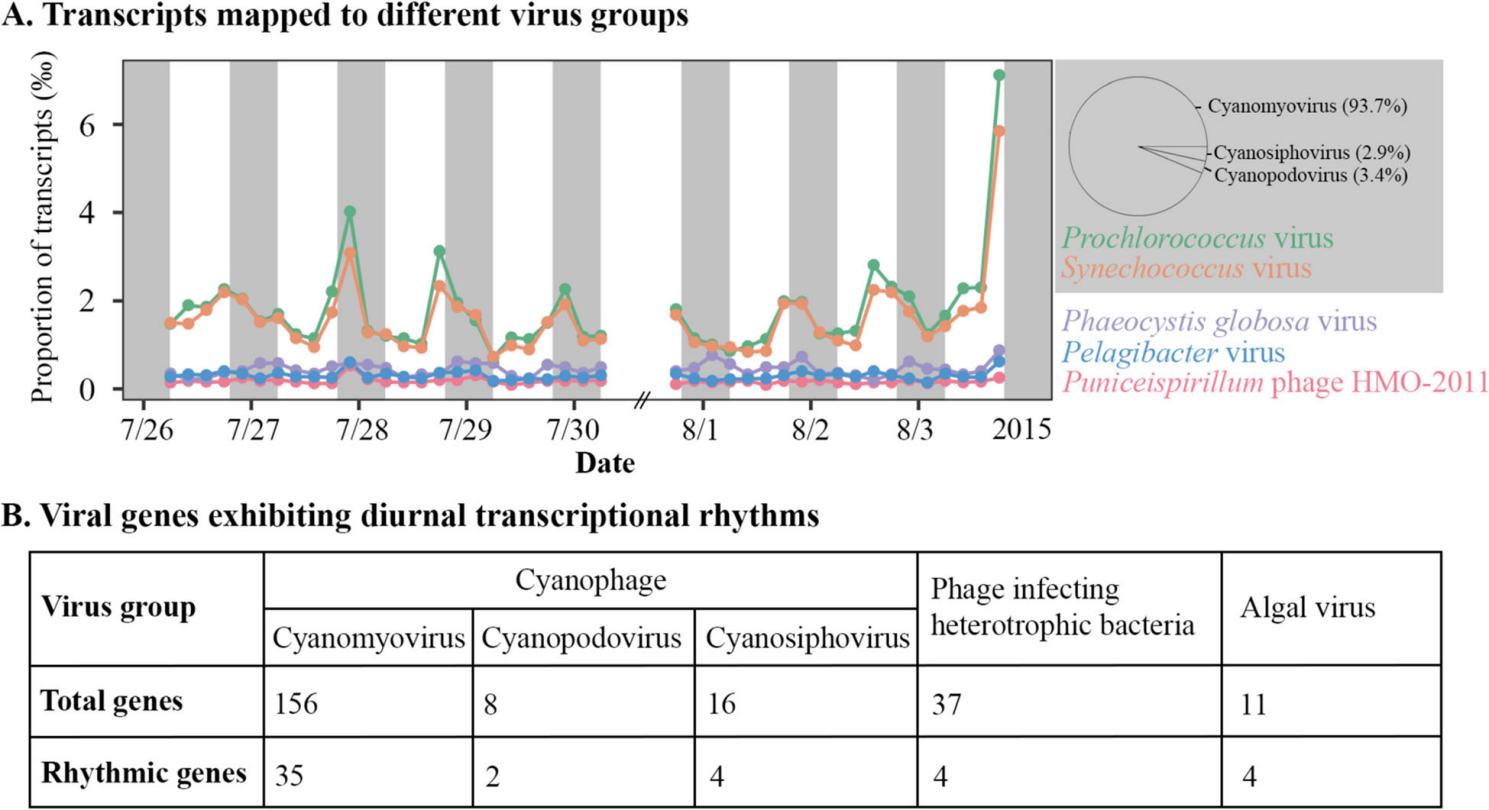
Diel transcript abundances of viral genes in the North Pacific Subtropical Gyre. **a** Every 4 h over 8 days (July 27–31 and August 1–3, 2015), the transcript abundances of viral genes at each sampling time point were normalized to the total non-rRNA transcripts that were aligned to an in-house microbial protein database. The transcript proportions of the five most abundant virus groups are shown. Grey bars indicate night periods. The pie chart on the right shows the average transcript proportions of cyanomyovirus, cyanopodovirus, and cyanosiphovirus relative to the total cyanophage transcripts. **b** Numbers of total genes and genes with diurnal transcriptional rhythms are shown for cyanophages, phages infecting heterotrophic bacteria, and algal viruses (see Supplementary Table 1 for details)

To test whether individual viral genes showed diurnal transcriptional rhythms, we reanalyzed the metatranscriptomic data from Aylward et al. [1] by mapping transcripts to viral genes (see “Materials and methods” section). Of the total transcripts in the microbial cellular size fractions, transcripts mapped to *Prochlorococcus* and *Synechococcus* phages were the most abundant viral transcripts, followed by those from *Phaeocystis globosa* viruses, pelagiphages, and *Puniceispirillum* phage HMO-2011 (Fig. 1a). Total cyanophage transcripts in the field populations increased during the day and decreased at night (Fig. 1a), which was similar to what we have observed in laboratory cultures and likely explained by diel changes in cyanobacterial photosynthesis [24]. After diel periodicity test (see “Materials and methods” section; Supplementary Table 1), 41 out of 180 cyanophage genes (23%) were found to have diurnal transcriptional rhythms (Fig. 1b and Supplementary Figures 1A, B, C). In addition, diurnal transcriptional rhythms were shown for 4 out of 37 genes (11%) of phages infecting heterotrophic bacteria, and 4 out of 11 genes (36%) of algal viruses (Fig. 1b and Supplementary Figures 1D, E). Therefore, our analyses revealed diurnal transcriptional rhythms of genes in various virus groups in the North Pacific Subtropical Gyre.

### Cyanophage genes with diurnal transcriptional rhythms

Cyanophage transcripts were mainly from cyanomyoviruses (93.7%), with a small portion from cyanopodoviruses and cyanosiphoviruses (Fig. 1a). In cyanomyoviruses, cyanopodoviruses, and cyanosiphoviruses, 35, 2, and 4 periodically expressed genes were identified, respectively (Fig. 1b). In cyanomyoviruses (Supplementary Table 1), the periodically expressed genes encode proteins for unknown function (16 genes), nucleic acid metabolism (9 genes), and phage capsid assembly (4 genes). Six auxiliary metabolic genes [4, 48, 49] were also found to be periodically expressed (Supplementary Table 1). These genes are involved in photosynthetic electron transport (plastoquinol terminal oxidase and NAD synthetase), central carbon metabolism (UDP-glucose 4-epimerase and glycerol-3-phosphate cytidylyltransferase), and stress responses (heat shock protein IbpA and P-starvation inducible protein PhoH) (Supplementary Table 1). In cyanopodoviruses, the two periodically expressed genes were both related to nucleic acid metabolism (Supplementary Table 1). In cyanosiphoviruses, two periodically expressed genes were related to nucleic acid metabolism and the other two related to capsid assembly (Supplementary Table 1).

**Fig. 2:**
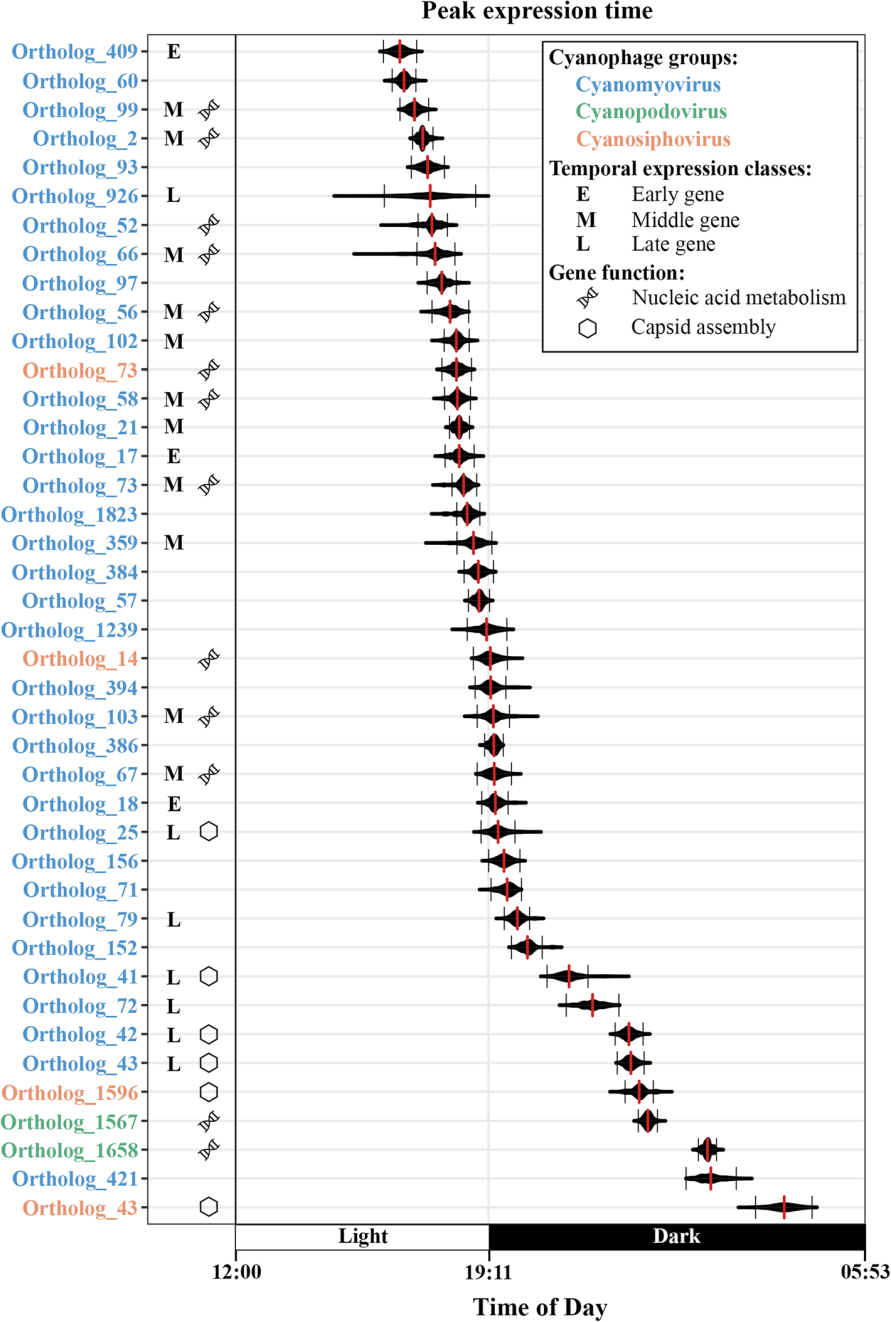
Peak expression times of periodically expressed cyanophage genes. The jackknife distributions of the estimated peak expression times of each gene are shown in violin plots. The red vertical line in each violin plot shows the peak expression time of each gene estimated from all data points and the two black vertical lines indicate the 90% confidence interval of the peak expression times estimated using the jackknife method from partial data points (Supplementary Figure 2). The color of the ortholog name denotes different cyanophage groups: blue for cyanomyovirus, green for cyanopodovirus, and orange for cyanosiphovirus. On the right of each ortholog name, E, M, L indicates early, middle, and late temporal expression classes, respectively. The double helix symbol indicates genes involve in nucleic acid metabolism and the hexagon symbol indicates genes involved in phage capsid assembly

It has been identified using cultured cyanomyoviruses that nucleic acid metabolism genes belong to middle genes and capsid assembly genes belong to late genes [10, 21]. Interestingly, in cyanomyovirus field populations, most genes related to nucleic acid metabolism showed peak transcript abundances before sunset (local time 19:11), while genes related to capsid assembly all peaked after sunset (Fig. 2). Similarly, in cyanosiphovirus field populations, the peak expression times of nucleic acid metabolism genes were also earlier than those of capsid assembly genes (Fig. 2).

### Peak expression times of early, middle, and late genes in cyanomyovirus field populations

**Fig. 3:**
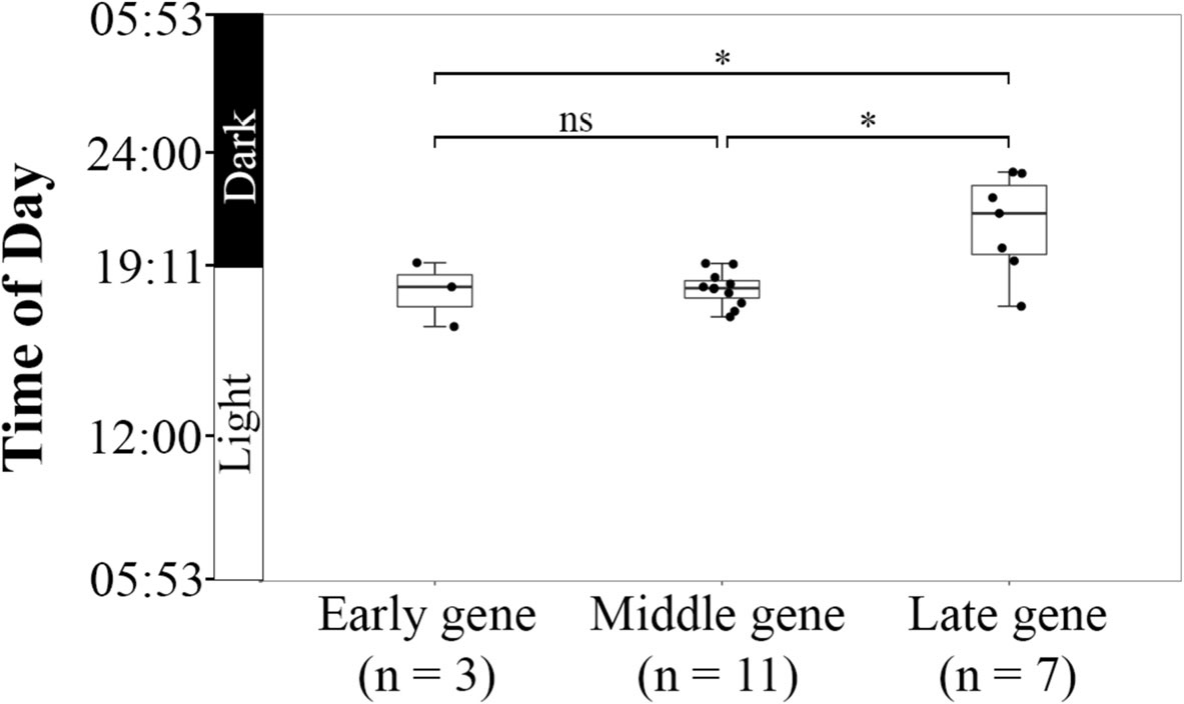
Comparison of the peak expression times of early, middle, and late genes in cyanomyovirus field populations. As shown in Fig. 2, the periodically expressed cyanomyovirus genes are classified as early (3 genes), middle (11 genes), and late genes (7 genes) according to the temporal expression patterns of their orthologs in cultured cyanophages. The peak expression times of early, middle, and late genes in cyanomyovirus field populations are shown in box and whisker plots. The bottom and top of a box indicate the first and third quartiles, respectively. The band inside a box indicates the median and the ends of a whisker represent the upper and lower extremes. Black dots indicate the estimated peak expression times of cyanomyovirus genes. The peak expression times of early, middle, and late genes are compared using the two-tailed Student’s test and the *p* values are indicated on top of the plots (*indicates *p* < 0.05 and ns indicates no significance)

We further tested whether the peak expression times of cyanomyovirus genes in the field populations correlated with the temporal expression patterns of their orthologs in cyanomyovirus laboratory cultures. Currently, the temporal expression patterns of cyanomyoviruses P-SSM2 [21], P-HM2 [47], P-TIM40, and Syn9 [10] have been characterized. In these laboratory experiments, cyanophage infection was synchronized so that the infecting cyanophages started the transcriptional program at the same time and the peak expression times of individual viral genes can be distinguished in bulk cultures. We were able to group 21 out of the 35 periodically expressed cyanomyovirus genes of field populations into early (3 genes), middle (11 genes), and late (7 genes) genes according to the conserved temporal expression patterns of their orthologs in the four cultured cyanomyoviruses (Fig. 2). The peak expression times of early and middle genes were not significantly different (Fig. 3). One potential explanation for this finding is that the 90% confidence interval of the calculated peak expression times of the periodically expressed genes is ~ 38 min (Supplementary Figure 2F) and may not be able to resolve smaller differences in expression time between the early and middle genes. Nevertheless, the peak expression times of the 7 late genes were significantly later than those of early and middle genes, suggesting that the temporal expression patterns of cyanomyovirus field populations followed those of synchronized cyanophage laboratory cultures [5, 10, 21, 47].

## Discussion

In this study, we reanalyzed the metatranscriptomic data from microbial cellular size fractions collected in the North Pacific Subtropical Gyre [1]. By mapping transcripts to individual viral genes, we identified 49 viral genes with diurnal transcriptional rhythms, including 41 genes from cyanophages, four from phages infecting heterotrophic bacteria, and four from algal viruses (Fig. 1b). For the periodically expressed cyanomyovirus genes, we found that the peak expression times of late genes were significantly later than those of early and middle genes (Fig. 3). In cyanophage laboratory cultures, the peak expression times of early, middle, and late genes were distinguished when cyanobacterial cells were infected at the same time [10, 21, 22, 46]. Therefore, the much later peak expression times of late genes in cyanomyovirus field populations suggest that cyanophage infection of cyanobacteria is synchronized in the North Pacific Subtropical Gyre.

Although our results provided evidence that cyanophage infection is synchronized in the North Pacific Subtropical Gyre, we were unable to use metatranscriptomic data to estimate the percentage of cyanophages that underwent synchronized infection. Future isolation-based studies and single-cell transcriptomic analyses are needed to measure synchronized and unsynchronized infections in the field populations.

Besides phages infecting *Prochlorococcus* and *Synechococcus*, we also identified periodically expressed genes from viruses infecting algae and heterotrophic bacteria. It is well known that marine algae exhibit circadian rhythms of growth [14] and gene expression [31]. Thus, similar to cyanophages [24], gene expression of viruses infecting *Aureococcus anophagefferens*, *Chrysochromulina ericina*, and *Phaeocystis globosa* (Supplementary Figures 1E) might also be regulated by the photosynthetic activity of algal host cells. Consistent with previous studies [1, 51], we identified rhythmic gene expression from a SAR11 virus *Pelagibacter* phage HTVC008M, and also from bacteriophages infecting enterobacteria *Cronobacter sakazakii* and *Klebsiella* (Supplementary Figure 1D). Diurnal transcriptional rhythms of heterotrophic bacteria were observed in the North Pacific Subtropical Gyre and were thought to be caused by rhythmic release of organic compounds from *Prochlorococcus* cells [32]. Therefore, the transcriptional rhythms of viruses infecting heterotrophic bacteria may also be due to rhythmic pulses of organic compounds. It should be noted that the periodically expressed genes from algal and bacterial viruses were identified by our metatranscriptomic analysis and their expression patterns need to be verified in future laboratory experiments.

Our metatranscriptomic analyses showed that the cyanomyovirus late genes mostly had their peak expression times in the dark period of a day and at a much later time than those of early and middle genes (Fig. 3). Our results not only suggest that cyanophage infection is synchronized to the light-dark cycle, but also hint at synchronized lysis of *Prochlorococcus* cells at night, which is consistent with the highest abundance of cyanophages at night in the Indian Ocean [6]. Our results help explain the light-dark synchronized mortality of *Prochlorococcus* cells in surface waters, which also reaches a peak at night [37]. Synchronized infection of *Prochlorococcus* by cyanophages may influence carbon cycling in the world’s oceans by synchronized release of dissolved organic matter to the marine food web during daily light-dark cycles.

## Conclusions

To demonstrate the synchronization of cyanophage infection in marine environments, our current study builds upon a previous metatranscriptomic dataset collected by Aylward et al. in the North Pacific Subtropical Gyre. By mapping metatranscriptomic reads to individual viral genes, we identified periodically expressed cyanophage genes. We also found that late genes (7 genes) in cyanophage field populations had a significantly later average peak expression time than those of early and middle genes, suggesting that cyanophage infection of *Prochlorococcus* is synchronized in the surface oceans. Our results are consistent with the light-dark synchronized population dynamics of *Prochlorococcus* cells in the oceans and have implications for *Prochlorococcus*-driven organic carbon production in the marine food web.

## Materials and methods

### Mapping metatranscriptomic reads to viral genes and orthologs

In the study conducted by Aylward et al. [1], microbial communities from > 0.2 μm size fractions were collected at 15 m depth in the North Pacific Subtropical Gyre every 4 h from July 26 to July 31 and from August 1 to August 3 2015. The metatranscriptomic data generated from these samples were downloaded from the NCBI Sequence Read Archive under accession number PRJNA358725.

Following the published protocols [2], downloaded sequences were quality-filtered and aligned to an in-house protein database. In brief, adaptor sequences and low-quality regions were trimmed using Trim_Galore (https://github.com/FelixKrueger/TrimGalore). Reads with low complexity and low quality were removed using PRINSEQ [39]. SortMeRNA [18] was then used to filter out rRNA reads based on alignment to the SILVA rRNA database (release 132) [35] and the Rfam 5S rRNA database [29]. After the removal of rRNA reads, the remaining transcripts were aligned to the in-house microbial protein database using LAST [17] with a bit score cutoff of 50. The microbial protein database included RefSeq Archaea, RefSeq Bacteria, RefSeq Protozoa, RefSeq Plant, and RefSeq Viral (RefSeq release 89), together with proteins of several additional cyanophages that were not in the RefSeq database during our analyses [8, 19, 34, 42]. Ambiguous sequences were discarded without further analysis if they were aligned equally well to proteins of multiple virus groups or to both a virus and its host.

Transcripts mapped to cyanophage genes were binned into cyanophage orthologs. When our study was conducted, there were 93 sequenced genomes of cyanophages in public databases that infect *Prochlorococcus* and *Synechococcus*. The gene orthologs (Supplementary Table 2) of the 93 cyanophage genomes were constructed using ProteinOrtho5 [20]. Transcripts with best hits to multiple orthologs were discarded. For viruses other than cyanophages, transcripts were not binned into orthologs, since there were not many sequenced genomes to construct gene orthologs.

### Identification of periodically expressed genes

To identify periodically expressed genes, the non-parametric RAIN package [45] was implemented in R [36] to test whether there is a 24-h rhythm in the transcript abundances of viral genes. The periodicity test was first conducted for cyanophage orthologs with at least 44 transcripts during the sampling period (an average of one transcript per sampling time point). The periodicity test was also conducted for genes of other virus groups with at least 44 transcripts. In the periodicity test, the transcript number of a gene/ortholog at each time point was normalized to the total non-rRNA transcripts that can be aligned to the in-house microbial protein database. Following a published protocol [1], a standard FDR correction [3] was applied to the *p* values generated by the RAIN package to remove false positives. The FDR-corrected *p* values under 0.1 denote significant periodicity and are shown in Supplementary Table 1.

### Peak expression times of periodically expressed genes

The average peak expression time of each periodically expressed viral gene during the sampling period was estimated based on the regression to a sinusoidal function. The Poisson log-linear regression to Eq. 1 was conducted on the transcript number at each time point to generate a best fit. The offset of the regression is the total number of transcripts with significant hits to proteins in our database. In Eq. 1, *x*_t_ represents the number of transcripts aligned to each gene at time *t*, and *α* and *β* were used to calculate the amplitude ($\sqrt{\alpha^2+{\beta}^2}$) and the phase shift ($\frac{12}{\pi }{\tan}^{-1}\frac{\beta }{\alpha }$) of the sinusoidal function. The significance of the fit was assessed using the chi-squared test as described previously [31] (implemented using the anova.glm function in R) [36]. The FDR corrected [3] *p* value from the chi-squared test under 0.1 implies a significant fit, and all periodic genes show significant fits (Supplementary Table 1). Based on the best-fit equation, the peak expression time (*t*_peak_) of each gene was calculated from Eq. 2.

1 $$ {x}_t=\upalpha \mathrm{cos}\left(\frac{2\pi }{24}t\right)+\upbeta \sin \left(\frac{2\pi }{24}t\right) $$

2 $$ {t}_{\mathrm{peak}}=6-\frac{\pi }{12}{\tan}^{-1}\left(\frac{\alpha }{\beta}\right) $$

The confidence interval of the estimated peak time for each gene was assessed using the delete-2 jackknife method [12], which is a classic re-sampling method that has been widely used in various biological systems [26, 28, 41, 44, 50]. In the delete-2 jackknife analysis, subsamples with 42 time points (2 time points deleted) were randomly drawn from the original 44 time points (totally 946 subsamples). The peak expression time of each subsample was calculated using the regression method mentioned above. With the peak expression times of the 946 subsamples, the 5% and 95% quantiles were calculated to determine the 90% confidence interval of the estimated peak expression time for each gene (Supplementary Figure 2). The 90% confidence intervals for the 49 periodic genes range from 21 to 104 min, with a median of 38 min (Supplementary Figure 2F and Supplementary Table 1).

## Supplementary information

**Additional file 1: Supplementary Figure 1.** Diel transcript abundances of periodically expressed viral genes in the North Pacific Subtropical Gyre. **Supplementary Figure 2.** Peak expression times of periodically expressed viral genes.

**Additional file 2: Supplementary Table 1.** Periodically expressed viral genes. **Supplementary Table 2.** Cyanophage protein orthologs.

## Data Availability

We reanalyzed published metatranscriptomic data [1]. Source data generated from the current study and the custom codes used for analyses are available on request from the corresponding author.
