# Correction to: Temporal transcriptional patterns of cyanophage genes suggest synchronized infection of cyanobacteria in the oceans

**DOI:** 10.1186/s40168-020-00891-0

**Published:** 2020-07-16

**Authors:** Yue Chen, Qinglu Zeng

**Affiliations:** 1 Department of Ocean Science, The Hong Kong University of Science and Technology, Clear Water Bay, Hong Kong, China; 2 Division of Life Science, The Hong Kong University of Science and Technology, Clear Water Bay, Hong Kong, China; 3 HKUST Shenzhen Research Institute, Shenzhen, China; 4 Hong Kong Branch of Southern Marine Science and Engineering Guangdong Laboratory (Guangzhou), The Hong Kong University of Science and Technology, Clear Water Bay, Hong Kong, China

**Correction to: Microbiome 8, 68 (2020)**

**https://doi.org/10.1186/s40168-020-00842-9**

The below statement in the Background of the original article [1] was incorrect. Individual viral genes with diel transcriptional rhythms were in fact listed in the supplementary dataset S05 (Sheet 2) of Aylward et al. 2017 [2]. This statement therefore needs to be corrected as outlined below. This correction does not affect the results and conclusions.

The original text:

“However, it was not shown in this study whether individual viral genes in a scaffold show diurnal transcriptional rhythms, as the combined transcripts mapped to all the genes in a scaffold were used for diel periodicity analyses ([1] reference [2] in this correction).”

Corrected text:

Several viral genes in the scaffolds also showed diurnal transcriptional rhythms [2], but it remains to be determined whether the peak expression times of early, middle, and late cyanophage genes are different.
