# Supplementary material for: Temporal transcriptional patterns of cyanophage genes suggest synchronized infection of cyanobacteria in the oceans
Source: Microbiome. 2020 May 19;8:68. doi: 10.1186/s40168-020-00842-9 (PMC7238727; doi:10.1186/s40168-020-00842-9)
Supplement: Supplementary file 2 — Additional file 1: Supplementary Figure 1. Diel transcript abundances of periodically expressed viral genes in the North Pacific Subtropical Gyre. Supplementary Figure 2. Peak expression times of periodically expressed viral genes. [file 40168_2020_842_MOESM1_ESM.pdf]

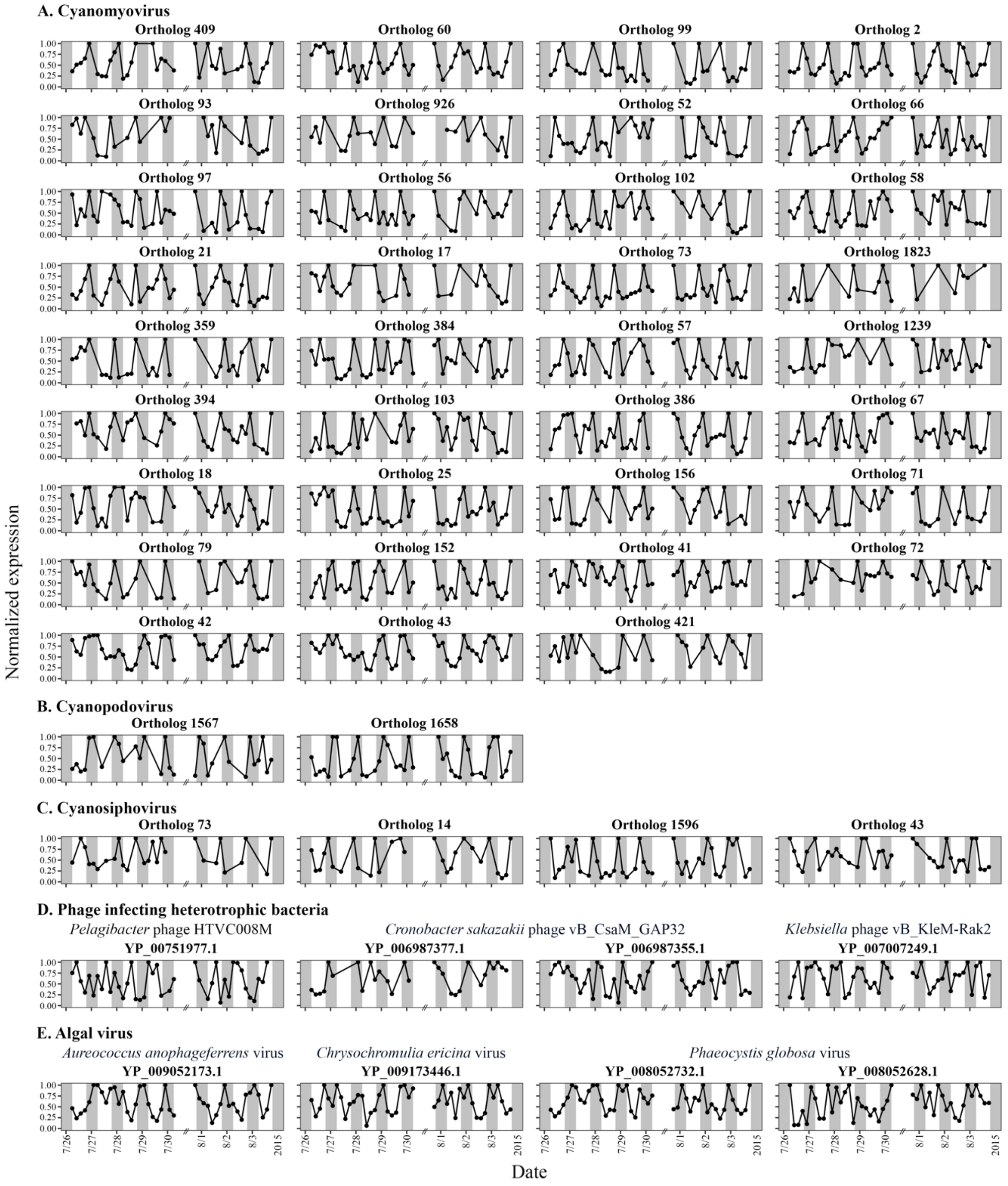

Supplementary Figure 1. **Diel transcript abundances of periodically expressed viral genes in the North Pacific Subtropical Gyre**

Periodically expressed genes are identified in different virus groups: cyanomyovirus (**A**), cyanopodovirus (**B**), cyanosiphovirus (**C**), phage infecting heterotrophic bacteria (**D**), and algal virus (**E**). At each time point, the transcript abundance of a gene was normalized to the total non-rRNA transcripts with significant hit to the in-house protein database and then was further normalized to the daily maximum level. The normalized expression levels of viral genes, ranging from 0 (no expression) to 1 (daily maximum expression), were plotted in line charts, with the sampling times shown on the bottom of the charts. Grey bars in the charts indicate night periods.

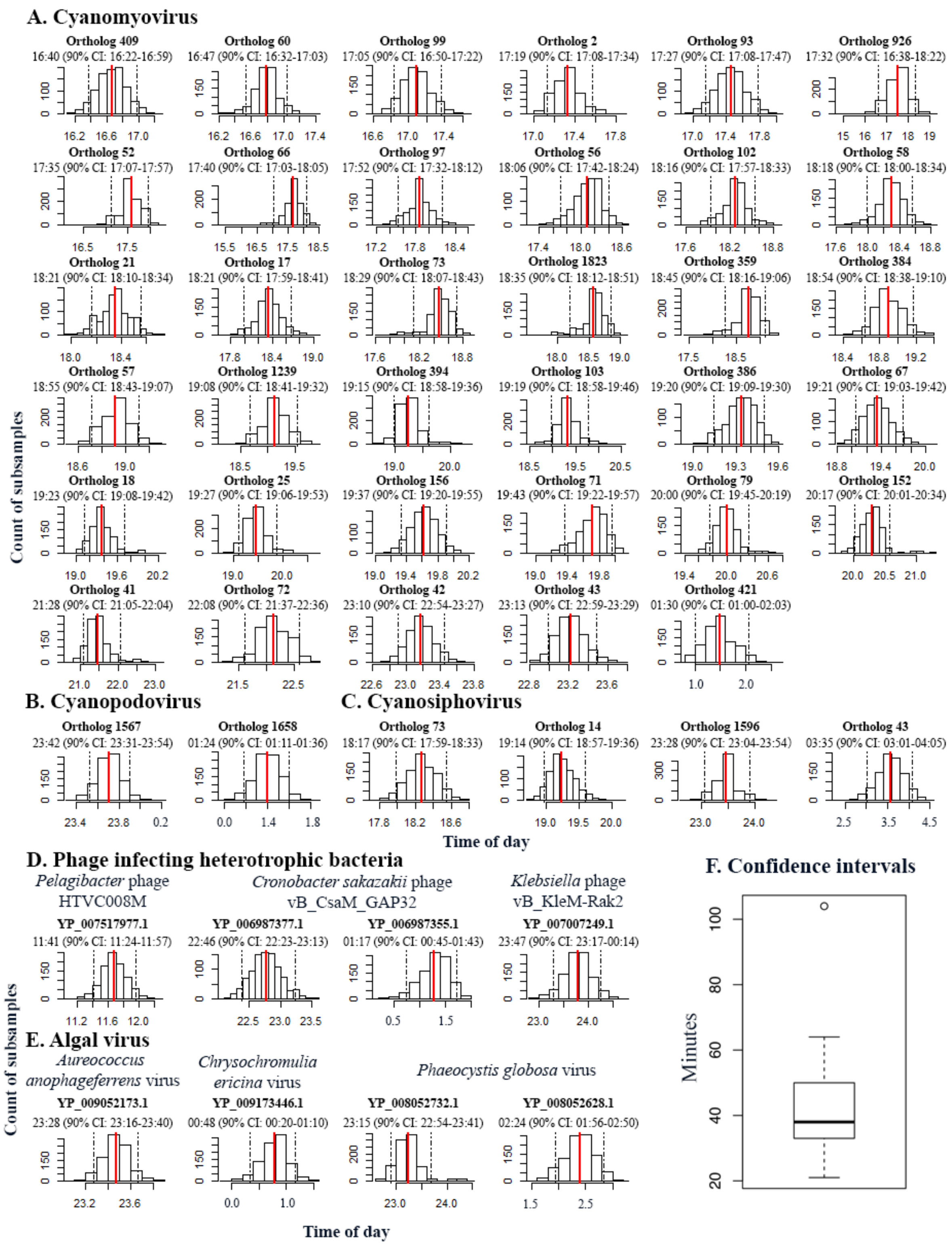

Supplementary Figure 2. **Peak expression times of periodically expressed viral genes**

The distributions of the estimated peak expression times generated by the jackknife method are shown for the periodically expressed viral genes: cyanomyovirus (**A**), cyanopodovirus (**B**), cyanosiphovirus (**C**), phage infecting heterotrophic bacteria (**D**), and algal virus (**E**). In each histogram, the two dashed lines represent the 5% quantile (left line) and 95% quantile (right line) of the 946 peak expression times that were estimated using partial data points. The red line indicates the peak expression time calculated from all data points. Above each

histogram, the peak expression time and the 90% confidence interval (90% CI, in parentheses) are shown. The distribution of the 90% confidence intervals of the peak expression times of all periodically expressed viral genes is shown in a boxplot (**F**). The bottom and top of the box indicate the first and third quartiles, respectively. The band inside the box indicates the median and the ends of the whisker represent the upper and lower extremes. The circle above the boxplot represents an outlier.
